# The Aging of Iron Man

**DOI:** 10.3389/fnagi.2018.00065

**Published:** 2018-03-12

**Authors:** Azhaar Ashraf, Maryam Clark, Po-Wah So

**Affiliations:** ^1^Institute of Psychiatry, Psychology and Neuroscience, Department of Neuroimaging, King's College London, London, United Kingdom; ^2^Department of Cell and Developmental Biology, University College London, London, United Kingdom

**Keywords:** brain iron, iron regulation, iron overload, aging, neuroinflammation, neurodegenerative diseases, Alzheimer's disease, Parkinson's disease

## Abstract

Brain iron is tightly regulated by a multitude of proteins to ensure homeostasis. Iron dyshomeostasis has become a molecular signature associated with aging which is accompanied by progressive decline in cognitive processes. A common theme in neurodegenerative diseases where age is the major risk factor, iron dyshomeostasis coincides with neuroinflammation, abnormal protein aggregation, neurodegeneration, and neurobehavioral deficits. There is a great need to determine the mechanisms governing perturbations in iron metabolism, in particular to distinguish between physiological and pathological aging to generate fruitful therapeutic targets for neurodegenerative diseases. The aim of the present review is to focus on the age-related alterations in brain iron metabolism from a cellular and molecular biology perspective, alongside genetics, and neuroimaging aspects in man and rodent models, with respect to normal aging and neurodegeneration. In particular, the relationship between iron dyshomeostasis and neuroinflammation will be evaluated, as well as the effects of systemic iron overload on the brain. Based on the evidence discussed here, we suggest a synergistic use of iron-chelators and anti-inflammatories as putative anti-brain aging therapies to counteract pathological aging in neurodegenerative diseases.

## Introduction

Iron dyshomeostasis is a molecular signature associated with aging, accompanied by progressive decline in cognitive processes. The transition element, iron, is the most abundant metal in the brain and performs pleiotropic functions including neurotransmitter synthesis, myelination of neurons and adenosine triphosphate (ATP) synthesis (Ward et al., 2014). Iron undergoes dynamic redox coupling through reversible oxidation/reduction of ferrous iron (Fe^2+^) and ferric iron (Fe^3+^). Hydrogen peroxide is often generated in the cell, especially as it is a by-product of normal mitochondrial respiration and can react with Fe^2+^ to produce hydroxyl free radicals in the Fenton reaction and increase reactive oxygen species (ROS), enhancing oxidative stress and eventually lead to cell damage (Kruszewski, 2003). However, whilst readily demonstrated in experimental systems, the Fenton reaction has been hard to observe *in vivo*, particularly due to the difficulties in measuring free radical reactions *in vivo*. Nonetheless, knowledge of free radical chemistry *in vitro* combined with accumulating circumstantial evidence e.g., use of iron chelators (see below), suggest a role for Fenton chemistry in free radical-mediated pathology. Consequently, the homeostatic neuroregulation of iron is tightly controlled for this reason, this “goldilocks” mineral can lead to perturbations in neuronal function if deficient or in excess. The aim of the present review is to focus on the age-related alterations in iron metabolism from a cellular and molecular biology perspective, alongside genetics, and neuroimaging aspects, in man and rodent models. Herein, therapeutic interventions are proposed to maintain healthy brain aging and attenuate neurodegenerative disease.

## Systemic iron homeostasis

**Figure 1:**
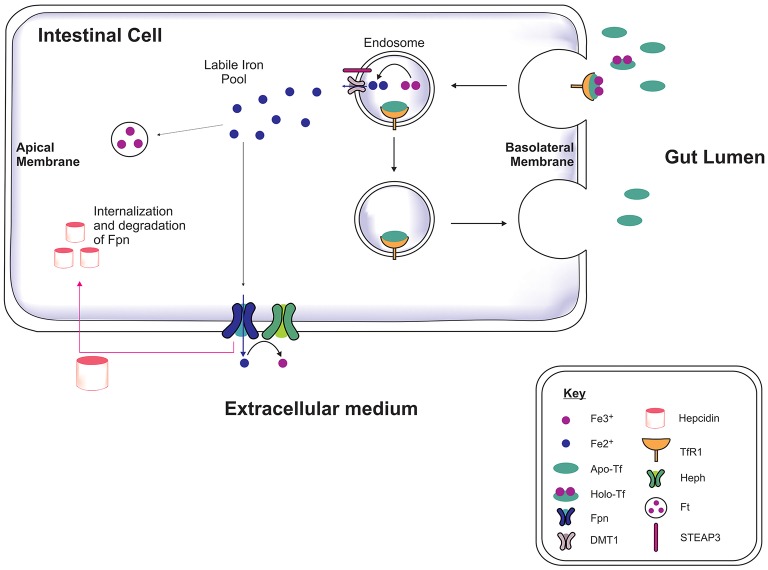
Systemic iron regulation. Iron-loaded apo-transferrin (Apo-Tf) forms holo-Tf and binds to Tf receptor-1 (TfR1) on the intestinal membrane and the holo-Tf/TfR1complex is endocytosed via clathrin-coated pits (Lambe et al., 2009; Duck and Connor, 2016). STEAP3 reduces the ferric iron (Fe^3+^) to ferrous iron (Fe^2+^) in the endosome and Fe^2+^ is transported into cytosol by divalent metal transporter-1 (DMT1), contributing to the labile iron pool. The apo-Tf/TfR1 complex is recycled to the cell membrane, where apo-Tf is released to bind to circulating plasma Fe^3+^. The excess iron is either sequestrated by ferritin or exported out of the cell by ferroportin (Fpn), in association with hephaestin (Heph). Hepcidin binds to Fpn mediating Fpn internalization and degradation, thereby preventing dietary iron absorption, dependent on the body iron status (Ganz, 2013).

Iron is acquired from the diet in the form of heme and iron salts and initially absorbed by the gut (Figure 1) which functions as a key modulator of iron concentration in the body (Ganz, 2013; Duck and Connor, 2016). Divalent metal transporter-1 (DMT1) and ferroportin-1 (Fpn) transport iron across the duodenal mucosa into the bloodstream (Fuqua et al., 2012). Fpn-mediated efflux is facilitated by a membrane-bound ferroxidase, hephaestin, which oxidizes Fe^2+^ to Fe^3+^ (Steere et al., 2012). In the serum, two Fe^3+^ are bound by apo-transferrin (apo-Tf) to become holotransferrin (holo-Tf), and transported in the latter form throughout the body. Approximately 30% of circulating iron is holo-Tf (Aisen et al., 2001), while the remaining iron associates with citrate, ATP and ascorbate to form low molecular weight complexes in the blood (Qian and Shen, 2001; Ma et al., 2009). Hepcidin is a peptide hormone produced by hepatocytes when exposed to excess iron, inducing cellular degradation of Fpn, thereby attenuating duodenal iron uptake (Ganz and Nemeth, 2012). Cells often express the Transferrin Receptor-1 (TfR1) on the plasma membrane which on binding to holo-Tf forms a complex (Lambe et al., 2009) and is endocytosed into an endosome. Within the endosome Fe^3+^ is reduced to Fe^2+^ by six transmembrane epithelial antigen of protein 3 (STEAP3, a metalloreductase) and Fe^2+^ dissociates from transferrin (Tf). The Fe^2+^ is then exported to the cytoplasm via DMT1 while Tf is recycled. Cytosolic Fe^2+^ can be used for metabolism, e.g., for synthesis of mitochondria iron-sulfur proteins, or sequestered/stored by ferritin to prevent iron engaging in redox reactions leading to toxicity (Rouault, 2006; Duck and Connor, 2016).

## Iron entry into the brain

**Figure 2:**
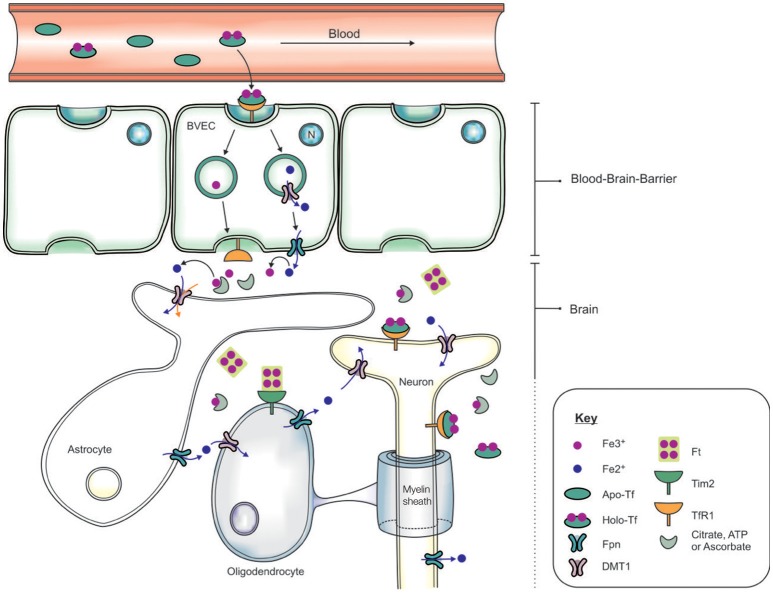
Schematic of the transportation of iron across the blood brain barrier (BBB) and into the brain. Apo-transferrin (Apo-Tf) binds to ferric iron (Fe^3+^) in the systemic circulation to form holo-Tf (Ke et al., 2005; Moos et al., 2007; Leitner and Connor, 2012). The BBB, formed by tight junctions between brain vascular endothelial cells (BVECs), limits transport of materials from the blood into the brain. Systemic holo-Tf binds to the Tf receptor-1 (TfR1) located on the luminal (blood) side (BVECs), and the newly formed holo-Tf/TfR1 complex is internalized into BVECs. Endosomal ferri-reductase catalyzes the reduction of Fe^3+^ to ferrous iron (Fe^2+^), enabling Fe^2+^ export into the cytosol from the endosome possibly through divalent metal transporter-1 (DMT1). At the abluminal (brain) side, the cellular Fe^2+^ enters the interstitium through an iron exporter, ferroportin, aided by rapid oxidation of Fe^2+^ to Fe^3+^ by ferroxidases (ceruloplasmin, hephaestin). The expression of polarized DMT1 in astrocytic foot-processes from close associations with BVECs, may facilitate rapid uptake of Fe^2+^ following their release into the perivascular space of the brain (Ward et al., 2014). Neurons can influx iron via a holo-Tf/TfR1-dependent uptake mechanism. In contrast, detectable levels of TfR1 are not expressed by either astrocytes nor oligodendrocytes. Tim-2, a ferritin receptor (Han et al., 2011), aids in oligodendrocyte iron uptake while non-Tf bound iron is taken up via DMT1.

The systemic circulation is physically separated from the brain parenchyma by two cellular barriers: the blood brain barrier (BBB) and the blood-cerebrospinal fluid barrier (BCSFB) (McCarthy and Kosman, 2015), and iron is transported across the barriers by an orchestra of molecular players. The BBB comprises brain microvascular endothelial cells (BVEC), astrocytes and pericytes which form stringent tight-junctions (Rouault and Cooperman, 2006), limiting access into the brain (Figure 2). The mechanism of iron entry into the brain comprises of two transmembrane steps: iron uptake into BVEC at the luminal (blood) side and iron efflux into the brain interstitial fluid at the abluminal (brain) side (Mills et al., 2010). Holo-Tf in the systemic circulation binds to luminal receptors (of BVEC) and the Holo-Tf/TfR1 complex internalized into endosomes (Hentze et al., 2004). Within the endosome, Fe^3+^ is reduced by a ferri-reductase, possibly duodenal cytochrome b (Dcytb) or Steap2, and liberated from Tf (De Domenico et al., 2008). The resulting Fe^2+^ ions are then exported from the endosome into the cytosol, likely via DMT1, although DMT1 expression and function in BVEC remains contentious. Cellular Fe^2+^ is released into the interstitial fluid through Fpn on the BVEC abluminal side, requiring re-oxidization of Fe^2+^ by ferroxidases such as hephaestin and/or extracellular ceruloplasmin (Ward et al., 2014; McCarthy and Kosman, 2015).

**Figure 3:**
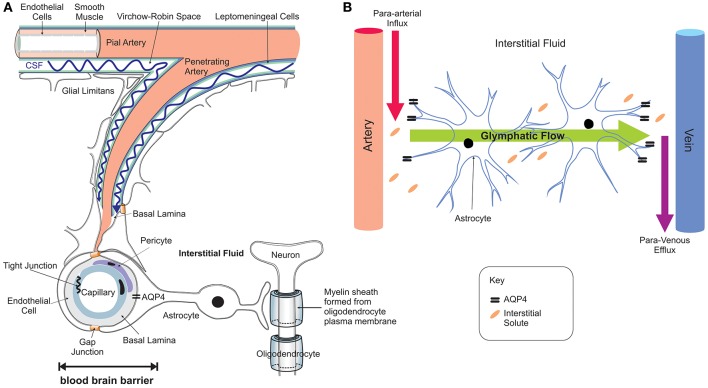
Neuro-vascular unit and the glymphatic pathway. The neuro-vascular unit **(A)** regulates transport into the brain and the glymphatic pathway enables communication between the microvasculature and neurons, with astrocytes acting as intermediators **(B)**. The CSF enters the brain parenchyma along paravascular spaces surrounding the penetrating arteries (Virchow-Robin space) exchanges with the brain interstitial fluid (Iliff et al., 2012, 2013) that bathes the brain (red arrow **B**; para-arterial CSF influx route). Interstitial solutes are subsequently cleared to paravascular spaces located in the vicinity of large caliber draining veins (purple arrow; para-venous interstitial fluid clearance route). The trans-parenchymal pathway (green arrow) allows convective bulk flow between the paravascular CSF influx and interstitial fluid efflux pathways facilitated by the exclusive perivascular astrocytic end-feet expressed aquaporin-4 (Aqp4) water channels (Nielsen et al., 1997; Mathiisen et al., 2010). This convective bulk flow mediates clearance of the interstitial solutes from the brain.

Iron entry into the brain may also occur through epithelial cells of the choroid plexus which constitute the BCSFB (Rouault et al., 2009). In contrast to the capillaries of the BBB, the choroid plexus consists of fenestrated capillaries that are readily crossed by holo-Tf to reach the choroidal epithelium (Brown et al., 2004) and iron crosses the choroidal epithelium to enter the ventricles. *In situ* hybridization revealed significant expression of TfR1, DMT1, Fpn, and ferroxidases (ceruloplasmin/hephaestin) in the choroid plexus (Dickson et al., 1985; Giometto et al., 1990; Wu et al., 2004; Rouault et al., 2009). This suggests that DMT1 is needed to release iron from the holo-Tf/TfR1 complex and iron is exported from the choroid epithelium to the CSF by Fpn, aided by the ferroxidases. Interestingly, the choroid plexus produces CSF that fills all four ventricles and bathes the interstitium of the central nervous system (CNS) (Rouault and Cooperman, 2006). It is important to emphazise that there is no diffusional barrier separating the ventricular CSF from the brain interstitial fluid. Once iron enters the interstitial fluid or ventricular CSF, it binds to Apo-Tf synthesized locally by the choroid plexus and oligodendrocytes to form holo-Tf (Leitner and Connor, 2012) and supplies CNS cells expressing TfR1. There is sparse data on Tf transport from the brain to the blood but apo- and holo-Tf may pass through the arachnoid villi and cribriform plate into the veins to enter the systemic circulation (Johansson et al., 2008). Diurnal variation of iron content in the ventral midbrain has been reported (Unger et al., 2009), reinforcing the notion that mechanisms exist for iron export from the brain to the systemic compartment. The recent discovery of the glymphatic system (Iliff et al., 2012; Papadopoulos and Verkman, 2013; Louveau et al., 2015; Trevaskis et al., 2015), a “lymphatic” system in the brain, may be one of the routes by which transition metals including iron are transported and cleared from the brain—through convective bulk flow of interstitial fluid, possibly aided by astrocytic aquaporin 4 channels (Figure 3). The mechanisms underlying the glymphatic system remain to be established.

## Brain iron homeostasis

**Figure 4:**
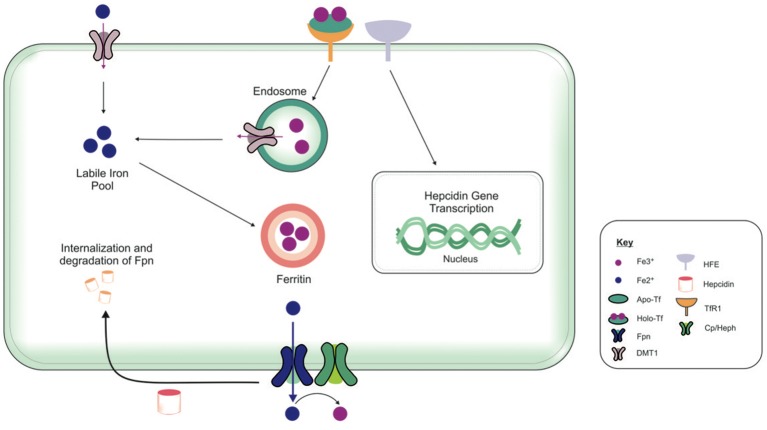
Overview of cellular metabolism of iron in the brain. Transferrin (Tf) laden with iron (holo-Tf) binds to the Tf receptor-1 (TfR1), and iron enters the cell by receptor-mediated endocytosis and into endosomes (Moos and Morgan, 2004; Moos et al., 2007). Iron can be released from endosomes into the cytosolic labile iron pool, from here, iron can either be utilized in cellular processes or stored in ferritin (Ward et al., 2014). Additionally, ferrous iron (Fe^2+^) may enter cells via divalent metal transporter-1 (DMT1) into the cytosolic labile iron pool, but only a relatively small amount of ferrous iron is transported by this way. Excess Fe^2+^ may also be exported from the cell by ferroportin (Fpn), aided by membrane-bound ferroxidase ceruloplasmin (only present in astrocytes) (De Domenico et al., 2007). Ceruloplasmin is not known to regulate the Fpn-mediated export but stabilizes Fpn. Ferroxidases (hephaestin and ceruloplasmin) in the circulation or the cell surface oxidize Fe^2+^ to ferric (Fe^3+^) iron and facilitates binding to apo-Tf to form holo-Tf for vasculature transport. Hepcidin in the extracellular space inhibits iron export by binding to Fpn and mediates Fpn internalization and degradation (Ganz and Nemeth, 2012). The extracellular iron status modulates the cellular hepcidin levels by interactions between TfR1 and HFE TfR1which in turn regulates cellular hepcidin levels via unknown mechanisms. The key proteins involved in peripheral and central iron homeostasis are similar. However, the brain has its own unique regulatory mechanisms, as it is isolated by the cellular barriers–the blood brain and blood-CSF barriers. The exact way in which the different brain cell types interact with one another to maintain iron homeostasis remains to be elucidated. Also, the mechanism underlying the cross-talk between the brain and the periphery to regulate global iron homeostasis is not fully characterized.

The brain comprises neurons and the glia, oligodendrocytes, astrocytes, and microglial, each with their specific complement of molecules in regulating their cellular iron content. Neurons can uptake both Tf-bound and non Tf-bound iron (via DMT1) from the interstitial fluid (Burdo et al., 2001). Iron is essential for axon myelination and oligodendrocytes only acquire non Tf-bound iron via the T-cell immunoglobulin and mucin domain (Tim-2), a ferritin receptor exclusively expressed in oligodendrocytes (Han et al., 2011), since TfR1 is not expressed by mature oligodendrocytes (Moos et al., 2007). Astrocytes on the other hand, express TfR1 but non Tf-bound iron is considered the main iron source for astrocytes *in vivo* (Moos and Morgan, 2004; Bishop et al., 2010) as TfR1 expression has only been reported in hippocampal astrocytic cultures (Pelizzoni et al., 2013). Astrocytic plasma membranes may express a ferri-reductase close to DMT1, to reduce Fe^3+^ to Fe^2+^, facilitating iron uptake (Bishop et al., 2010). Microglia phagocytose cells to recycle iron, with excess iron being stored with ferritin. Ferritin is the principal storage protein used to sequester iron in neurons and glia, and indeed in many cell types. The primary efflux iron transporter is Fpn, working in concert with ceruloplasmin to generate Fe^3+^ iron for cell export (Ward et al., 2014). Depending on iron status (iron overload or deficiency), hepcidin can accordingly regulate the expression of Fpn to increase or decrease the efflux of intracellular iron (Figure 4). Cellular iron levels are also modulated at the post-transcriptional (or translational) level by binding to the iron responsive elements (IREs) of mRNA of iron regulatory proteins (IRPs), e.g., ferritin, TfR1 (Ward et al., 2014). In the presence of surplus iron, IRPs remain inactive and do not bind to the IREs on the mRNAs of ferritin and TfR1, enhancing ferritin synthesis while TfR1 mRNA undergoes degradation by nucleases (Wallander et al., 2006; Anderson et al., 2012). On the other hand, in conditions of iron deficiency, the IRPs bind to the IREs on the mRNAs in the 3′-untranslated region (UTR) to prevent TfR1 mRNA degradation resulting in TfR1 synthesis. The binding of IRPs to the IREs in the 5′-UTR of ferritin mRNA prevents translation of ferritin and ferritin is not synthezised.

The cellular and molecular mechanisms operant in regulation of iron uptake, transport, and metabolism in the brain are intricate and intertwined. Disruption of these mechanisms leads to iron dyshomeostasis, which has become a common feature in neurodegenerative diseases (Ward et al., 2014). A plethora of iron homeostatic mechanisms appear to be affected during physiological aging and may explain the increased susceptibility of the aged brain to disease and why aging is the major risk factor in neurodegenerative diseases. However, octogenarians/centurions, or “super-agers” are known, who remain disease—and disability-free throughout aging. Thus, it is imperative to distinguish between normal and pathological aging to develop fruitful therapeutic targets. The mechanisms underlying iron perturbations in the brain are discussed with respect to normal aging and in neurodegeneration, including the relationship between iron dyshomeostasis, and neuroinflammation, and the effects of systemic iron overload on the brain.

## Age-associated iron deposition

### Humans

Based on human post-mortem analyses, total iron deposition is positively correlated with age with maximal levels achieved in the basal ganglia including the putamen, globus pallidus, and substantia nigra (Hallgren and Sourander, 1958; Connor et al., 1990, 1992, 1995). Meanwhile, the lowest concentrations were observed in the cerebral cortex, brainstem, and cerebellum (Zecca et al., 2004; Ramos et al., 2014).

At the cellular level, oligodendrocytes are the predominant glial cells enriched with iron, ferritin, and Tf in both gray and white matter. The concentration of iron and associated proteins remains constant in the oligodendroglia with age (Connor et al., 1990). Iron and ferritin immuno-reactive microglial and astroglial cells generally showed increased expression during aging in the cerebral cortex, cerebellum, hippocampus, amygdala, and basal ganglia. In contrast to the gray matter, where iron is confined to ferritin-positive protoplasmic astrocytes, Tf expression in fibrous astroglia was noted exclusively in the white matter, particularly in aged individuals. The paucity of staining of iron and related proteins in neurons implies a primary role for glial cells, particularly astrocytes and microglia, in altering iron metabolism during the process of ageing (Connor et al., 1990).

The distribution of TfR1 exhibits disparity with ferritin and iron distribution in the human brain. Autoradiography of TfR1 revealed increased density in the neocortex, with moderate intensities in the putamen and caudate nucleus. The lowest density of TfR1 was evident in the globus pallidus, substantia nigra, and white matter regions (Griffiths and Crossman, 1996). Conversely, immunohistochemical staining of TfR1 did not reveal a consistent staining pattern (Connor and Menzies, 1996). In the dentate gyrus of the hippocampus, many but not all neurons, were immuno-positive for TfR1. In the striatum, TfR1-immunoreactivity was evident in both neurons and astrocytes. The iron-rich deep cerebellar nuclei did not stain for TfR1 but oligodendrocytes prevalent in peri-neuronal positions did exhibit immuno-positivity. Furthermore, the white matter tracts revealed light staining and was distributed between oligodendrocytes and fibrous astrocytes. The difference in TfR1 and ferritin is unsurprising, considering iron induces ferritin expression but decreases TfR1 expression.

More detailed analysis on age-related iron deposition has been undertaken in neuromelanin pigmented neurons of substantia nigra and locus coeruleus. The substantia nigra exhibits a linear increase in total iron concentration and ferritin with aging, remaining constant in the locus coerulus, underscoring the importance of the former in neurodegenerative diseases (Zecca et al., 1996, 2001). Interestingly, Perl's staining demonstrated deposits of iron in glial cell population and neuromelanin-deficient neurons in the substantia nigra of healthy individuals (Zecca et al., 2004). On the contrary, reactive iron deposits were not observed in neurons expressing neuromelanin, indicative of effective iron-sequestration by neuromelanin (Zecca et al., 2004, 2008a). The concentration of iron-neuromelanin complexes also increases with age in neurons of the putamen, premotor cortex and cerebellum, demonstrating augmented iron mobilization during aging (Zecca et al., 2008a). It is important to note that binding of iron to neuromelanin is variable in neurons of different brain regions (Ward et al., 2014). Evaluating the mechanism further, excess iron is released from neuromelanin and augments oxidative stress in mitochondria, leading to mitochondrial impairment. Both superoxide dismutase and deferoxamine counteracted the increase in iron levels, confirming the involvement of iron in superoxide production (Shamoto-Nagai et al., 2006). This transition of a shift in function of neuromelanin from being protective to detrimental, may explain the selective vulnerability of aged dopamine neurons to PD (Ayton and Lei, 2014). Moreover, co-localization of iron and dopamine in the substantia nigra renders the region susceptible to oxidative stress (Hare et al., 2014). Mice harboring 6-hydroxydopamine lesions demonstrated iron and dopamine dyshomeostasis, leading to increased iron and dopamine product mediated oxidative stress, which contributes to neurodegeneration in PD.

### Rodents

In congruence with human studies, in rodents, the oligodendrocyte is also the principal cell involved in iron regulation (Todorich et al., 2009). Oligodendrocytes undergo a complex and finely tuned program of proliferation, migration, differentiation, and myelination to produce insulating sheath of axons (Bradl and Lassmann, 2010). Since iron is required as a co-factor for myelin synthesis, this contributes to why oligodendrocytes is the CNS cell with the highest intracellular iron content. As in humans, an increase in ferritin–and iron-staining in microglia was observed with aging in the rat hippocampus, basal ganglia, and cerebellum (Roskams and Connor, 1994; Focht et al., 1997). In contrast to humans, rat astrocytes are the only neuroglial cell that does not stain for iron or iron-regulatory proteins. Interestingly, myelin-deficient rats lacking a mature population of oligodendrocytes, exhibit altered cellular iron distribution with increased iron in astrocytes and microglia while Tf remained unchanged (Connor and Menzies, 1990; Todorich et al., 2009). Apparently in rodents, astrocytes may accumulate iron when oligodendrocytes are compromised. There are striking differences between human and rodent astrocytes that may help explain these discrepant findings (Oberheim et al., 2009). Briefly, distinct morphologic classes of GFAP-positive astroglial cells (namely interlaminar and varicose projection astrocytes) have been exclusively identified in adult human temporal cortex (Oberheim et al., 2009). Long processes of interlaminar astrocytes, characterized by their tortuous appearance span cortical layers I-IV, terminate in the neuropil or occasionally on the vasculature. In layers V-VI, polarized astrocytes with long processes characterized by varicosities penetrating delimited domains of neighboring astrocytes in a random fashion have been sparsely observed in humans but not in rodents. Protoplasmic astrocytes are the most abundant glial cells in the cortex of humans and rodents, many fold larger in humans than in rodent (Bushong et al., 2002; Ogata and Kosaka, 2002), with extensive overlapping processes and increased GFAP-immunoreactivity in their end feet. Similarly, fibrous astrocytes in human white matter are larger than in rodents (Oberheim et al., 2009).

To stir the complexity further, not all astrocytes are GFAP-positive, confirming their phenotypic diversity in both species. Using patch-clamp recordings and single-cell PCR reverse transcription, transgenic mice with GFAP promoter-controlled green fluorescent protein expression demonstrated two different hippocampal astrocyte populations. One had thin and truncated processes with attenuated GFAP immunoreactivity, while the other showed more intense GFAP expression and processes displayed intricate morphology with varied electrical properties (Matthias et al., 2003). Furthermore, positron emission tomography (PET) studies point to prevalent heterogeneous binding to mitochondrial translocator protein (TSPO) ligands in humans. Activated microglia and astrocytes overexpress TSPO in normal elderly and demented subjects, however, individuals can be classed as high or low (or mixed) affinity TSPO-binders depending on the rs6971 polymorphism on the TSPO gene (Owen et al., 2012).

In aged rats, neuronal iron revealed by Perl's staining was evident, particularly in the basal ganglia and hippocampus, in the absence of a concomitant increase in ferritin immunoreactivity in neurons (Benkovic and Connor, 1993). Similarly, increased iron deposition with aging in wild-type mice was associated with a decline in immuno-reactive ferritin suggestive of iron dysregulation (Walker et al., 2016). Also, Parkinson's disease (PD) subjects showed increased iron deposition in neurons with a concomitant decrease of ferritin-reactivity in the substantia nigra, compared to that in neurons of age-matched healthy aged humans (Connor et al., 1995). Iron dyshomeostasis with aging may be due to an increase in iron that is not associated with ferritin. As mentioned previously, excess iron may be sequestrated by neuromelanin, protecting neurons against toxicity, including from by-products of dopamine metabolism. However, neuromelanin-containing organelles may then accrue high amounts of iron and neurotoxin overload with aging and prime a neurodegenerative response (Zucca et al., 2015). For example, leakage, or release of iron-laden neuromelanin from degenerated neurons induces microglial activation, with activated microglia in turn promoting neuronal death via release of additional neuromelanin. This induces a self-propagating mechanism of neuroinflammation and neurodegeneration, contributing to the predisposition of elderly individuals to neurodegenerative diseases.

Consistent with human studies, mice demonstrated TfR1 immunoreactivity predominantly in neurons scattered throughout the brain in gray matter regions (Moos, 1996; Dickinson and Connor, 1998); strongest in cortical (particularly pyramidal) neurons, hippocampal (Ammon's horn and dentate gyrus) neurons and cerebellar Purkinje cells (Dickinson and Connor, 1998). TfR1 expression was virtually absent in astrocytes, oligodendrocytes and microglia (Moos, 1996; Dickinson and Connor, 1998). Summarizing the findings, iron-rich oligodendrocytes does not exhibit TfR1 immunoreactivity while neurons stained for TfR1 but showed poor iron staining in rodents (Mash et al., 1990).

## Mechanisms underlying iron dysregulation

### Cytosolic ferritin

The principle iron storage protein, ferritin, is comprised of heavy (H) and light (L) chain monomers which co-assemble to form heteropolymers of 24 subunits. Ferritin is able to carry up to 4,500 iron atoms to attenuate cytosolic and nuclear free labile iron pools (Harrison and Arosio, 1996). The H-chain subunit, owing to its ferroxidase activity, oxidizes Fe^2+^ to Fe^3+^ to enhance iron sequestration by ferritin (Muhoberac and Vidal, 2013). On the other hand, the L-subunit facilitates iron-core formation and has a greater storage capacity than the H-subunit. In the brain, H-chain ferritin predominates in neurons, while the microglial and astroglial cells express mostly L-chain ferritin. Conversely, oligodendrocytes contain both H- and L-subunits implying a requirement of this glial cell to store and rapidly mobilize iron for its cellular needs. The ratio of H- to L-chain is precisely regulated, with even a small addition of mutant L-chain adequate to induce dysfunction (Li et al., 2015). An autosomal dominant mutation in the L-ferritin gene observed in congenital neuroferritinopathy, a neurodegeneration disorder with brain iron accumulation (NBIA), reduces iron incorporation into ferritin leading to an increased labile iron pool, rendering the cell overtly sensitive to oxidative damage (Levi et al., 2005). Similarly, L-ferritin variants, 460InsA and 498InsTC in HeLa and SH-SY5Y neuroblastoma cells, respectively, show reduced ferritin functionality and stability (Cozzi et al., 2010). The increase in cytosolic Fe^2+^ augments oxidative stress and leads to increased protein (and lipid) oxidation, and may promote proteasomal impairment and ferritin aggregate formation. Ferritin aggregates have been observed to accumulate in the nucleus and cytoplasm of subjects with neuroferritinopathy (Vidal et al., 2004) and in transgenic mice harboring the 498InsTC mutation (Barbeito et al., 2009). Evidence suggests that the pathogenesis of neuroferritinopathy results from a combination of diminished iron storage function and augmented neurotoxicity (Lee et al., 2006).

### Ferritin aggregates

Autophagy is the dominant process degrading cytosolic ferritin and mitochondrial electron transport proteins in lysosomes, liberating iron, and increasing cytosolic iron levels (Yu et al., 2003). Protein aggregation is able to trigger autophagy (Grune et al., 2004; Williams et al., 2006), tempting the postulation that ferritin aggregates are a preliminary step to lysosomal uptake. Electron microscopy of ferritin aggregates reveal the presence of covalently-linked dimers, trimers, tetramers, extending to larger oligomers (Harrison and Gregory, 1965; Williams and Harrison, 1968; Munro and Linder, 1978; Linder, 2013). The mechanism underlying ferritin aggregation remains unclear, nevertheless, ferritin oligomers bind to microtubules in a variety of cell lines including mouse neuroblastoma cells *in vitro* (Hasan et al., 2005, 2006). The resultant microtubule-bound ferritin is 2.5 times richer in iron than the unbound ferritin fraction. Further, when mouse neuroblastoma become iron-deficient, ferritin oligomers localized to dense microtubule neurite shafts but were absent in the microtubule neurite-deficient tips. Microtubules appear to act as a scaffold for the cytoplasmic distribution of ferritin oligomers and aid transport of iron-rich ferritin, implicating involvement of microtubules in iron metabolism (Hasan et al., 2005, 2006). Moreover, the regional variability of iron in the brain may be explained by lysosomes containing a range of iron concentrations. More active lysosomes contain higher amounts of iron and are more susceptible to oxidative stress (Nilsson et al., 1997; Parente et al., 2012). This satisfactorily explains why lysosomes of certain cells are resistant to substantial oxidative stress while others succumb to apoptosis even in the presence of a relatively low degree of stress (Antunes et al., 2001). Further, treatment of lysosomes with deferoxamine protected against hydrogen peroxide induced lysosomal rupture and apoptosis (Kroemer and Jäättelä, 2005). This finding underscores the detrimental effect of redox-active iron on lysosomal stability via enhanced oxidative stress (Kurz et al., 2008).

### Ferroptosis

An emerging process that is beginning to take center stage is ferroptosis, a newly characterized form of iron-dependent cell death distinct from apoptosis and necrosis (Dixon et al., 2012; Yang and Stockwell, 2016; Angeli et al., 2017). Ferroptosis is triggered by pharmacological impairment of anti-oxidant systems involving glutathione and glutathione peroxidase (GPX4) (Stockwell et al., 2017).

The glutamate/cystine antiporter (${\text{x}}_{\text{c}}^{-}$) exports cellular glutamate in exchange for extracellular cystine. Once inside the cell, cystine is converted to cysteine, a precursor of the endogenous anti-oxidant, glutathione (Dixon et al., 2012). Erastin and sorafenib, trigger ferroptosis via inhibition of ${\text{x}}_{\text{c}}^{-}$, depleting glutathione and inactivating GPX4 (Yang et al., 2014; Stockwell et al., 2017). Ferroptosis may also be induced by administration of GPX4 inhibitors, RSL3 and ML162. GPX4 catalyzes potentially toxic lipid hydroperoxides to non-toxic lipid alcohols (Yang et al., 2016) and its inactivation, whether via glutathione depletion or direct GPX4 inhibition, induces lipid peroxidation/oxidative stress, and eventually cell death. Deferoxamine is able to prevent ferroptosis-induced cell death through quenching of excess iron (Murphy et al., 1989; Cao and Dixon, 2016). Furthermore, ferroptosis is regulated by genes involved in iron metabolism via the Tf/TfR1 complex, IRP-2, iron-sulfur cluster assembly enzyme, L-ferritin and H-ferritin (Dixon et al., 2012).

The role of iron in ferroptosis is further substantiated by holo-Tf but not apo-Tf, being essential for induction of ferroptosis (Gao et al., 2015). Using gene silencing, size exclusion fractionation and mass spectrometry, holo-Tf and glutamine were shown to be required for ferroptosis, especially in the presence of cystine deficiency due to impairment of the glutaminolysis pathway (Gao et al., 2015). Indeed, silencing TfR1 expression or administration of iron chelators inhibited cell death, underscoring the requirement of holo-Tf import for ferroptosis-induced cell death (Gao et al., 2015). In addition, inhibition of lipid peroxidation either through genetic ablation of Acyl-CoA synthetase long-chain family member 4 (ACSL4), or pharmacological inhibition by lipo-oxygenase inhibitors, ferrostatins or liproxstatins suppresses ferroptosis. Although the physiological role of ferroptosis and the mechanisms governing ferroptosis-induced cell death remain to be elucidated (Stockwell et al., 2017), four processes are thought to be critical for ferroptosis: (1) iron accumulation, (2) glutathione depletion, (3) lipid peroxidation, and (4) membrane insertion of phosphatidylethanolamines, all processes that enhance oxidative stress (Bertrand, 2017; Doll et al., 2017; Kagan et al., 2017). Genetic or pharmacological inhibition of any of these three processes is sufficient to prevent ferroptosis (Dixon et al., 2012; Cao and Dixon, 2016; Yang and Stockwell, 2016; Angeli et al., 2017). Ferroptosis-mediated cell death appears to involve synergistic interactions of iron toxicity, depletion of anti-oxidant levels, insertion of phosphatidylethanolamines and lipid membrane damage, leading to amplification of oxidative cell damage in neurodegenerative diseases (Bertrand, 2017; Doll et al., 2017; Kagan et al., 2017).

Preliminary evidence has shown that ferroptosis inhibition can decrease the incidence of acquiring PD (Brauer et al., 2015; Do Van et al., 2016; Guiney et al., 2017; Ito et al., 2017). This has been investigated in a human dopaminergic neuronal precursor derived cell line, LUHMES, which not only exhibits unique sensitivity to ferroptosis but also recapitulates several features of ferroptosis when exposed to erastin, including glutathione depletion (Do Van et al., 2016). Deferiprone (an iron chelator) and N-acetylcysteine (a precursor of glutathione synthesis) was able to rescue these cells from erastin-induced toxicity. The enhancement of erastin toxicity by elevated dopamine was also shown to be rescued by ferroptosis inhibitors (Do Van et al., 2016). Apparently, dopaminergic neurons are inherently vulnerable to ferroptosis and could offer an additional explanation to their heightened susceptibility to neurodegeneration (Ayton et al., 2014). Moreover, pre-treatment of mice with ferroptosis inhibitors was able to rescue the behavioral impairment and neuronal loss due to 1-methyl-4-phenyl pyridinium (MPP+) toxicity, an experimental model of PD (Do Van et al., 2016). Ferroptotic damage after ischemic stroke was observed in 12-month old tau-knockout mice which display elevated brain iron (Tuo et al., 2017). Normalization of iron levels by ceruloplasmin and amyloid precursor protein (APP) ectodomain, or preventing ferroptosis by liproxstatin-1, attenuated infarct size. On a similar note, tau-knockout mice developed age-dependent brain atrophy, iron accumulation and loss of nigral neurons, alongside cognitive deficits and Parkinsonism (Lei et al., 2012). These pathological and behavioral changes were rescued by oral treatment of tau-knockout mice with an iron chelator, clioquinol. Tau expression is an inhibitor of age-related brain iron accumulation and demonstrates antagonistic pleiotropy as tau suppression in youth is neuroprotective but exacerbates age-related neurotoxic iron accumulation (Tuo et al., 2017).

Heme toxicity can be expressed in a plethora of ways; via generation of superoxide and hydroxyl radicals; release of redox-active iron; depletion of cellular stores of NADPH and glutathione; and increased lipid peroxidation. Prolonged exposure to hemoglobin and Fe^2+^ induces ferroptosis in organotypic hippocampal slice cultures as confirmed by the efficacy of ferroptosis inhibitors (Hahl et al., 2013). Hemopexin, a glycoprotein, can bind heme with high affinity and induce expression of heme-oxygenase 1 (HO-1) to confer neuroprotection against heme and its downstream effects (Hahl et al., 2013). Ferrostatin-1, a ferroptosis inhibitor, attenuated hemoglobin-induced neuronal death and reduced iron deposition in organotypic hippocampal slices (Li et al., 2017). Similarly, ferrostatin-1 treated mice showed markedly improved neurological function after intracerebral hemorrhage, and other ferroptosis inhibitors (liproxstatin-1 and zileuton) effectively reduced neuronal death (Li et al., 2017). Hemoglobin also induced a dramatic increase in prostaglandin-endoperoxide synthase 2, previously observed in erastin analogs and RSL3-induced ferroptosis (Dixon et al., 2012; Yang and Stockwell, 2016), that was reversed by ferrostatin-1.

Autophagy may contribute to ferroptosis through selective degradation of ferritin (ferritinophagy) and increasing the labile iron pool, leading to accumulation of ROS and ultimately driving ferroptosis (Gao et al., 2016; Hou et al., 2016; Torii et al., 2016). The pathway is mediated by nuclear receptor co-activator 4 (NCOA4, autophagy cargo receptor) binding to ferritin in the autophagosome to deliver ferritin to the lysosome for degradation with the release of iron (Mancias et al., 2014). Genetic ablation of NCOA4 attenuated ferritin degradation and subsequent ferroptosis, while forced NCOA4 expression induced ferritin degradation and induced ferroptosis (Hou et al., 2016). The purpose of the autophagic degradation of ferritin (aka ferritinophagy) is to release iron during anemia for the crucial survival process of erythropoiesis (Mancias et al., 2015). The dysregulation of ferritinophagy may have detrimental consequences to cell function mediated by ferroptosis in pathological conditions.

### Lipofuscin

Lipofuscin is considered a hallmark of aging, showing a late accumulation that inversely correlates with longevity (Brunk and Terman, 2002; Terman and Brunk, 2004). Lipofuscin-associated iron sensitizes lysosomes to oxidative stress, destabilizing lysosome stability and inducing apoptosis via release of lysosomal contents (Brunk and Terman, 2002; Terman and Brunk, 2004). Similarly, chronic intracerebroventricular injections of ferrous ammonium sulfate in 3–4 months old male CFY white rats induced a 65% increase in the volume density of lipofuscin in the parietal cortex and increased cerebral iron (Nagy et al., 1985). Centrophenoxine (a cholinergic nootropic used as a dietary supplement but also to treat symptoms of AD) pre-treatment, not only prevented iron accumulation in rat cortical synaptosomes following ferrous ammonium sulfate injection into the CSF (Zs-Nagy et al., 1995) but also attenuated lipofuscin deposition (Roy et al., 1983). Ultrastructural analyses showed accumulation of lipofuscin granules associated with iron deposits, particularly enriched in the cerebellum and striatum of transgenic mice harboring a L-ferritin subunit mutation (498–499InsTc mutation) (Maccarinelli et al., 2015). Collectively, the data emphasizes that iron is sufficient to increase lipofuscin formation possibly by increased iron-induced lipid peroxidation. Hence, accumulation of iron-rich material, e.g., ferritin, resistant to cellular degradation may enhance formation of lipofuscin. Hemosiderin has been considered to be an unusually iron-rich form of lipofuscin (Terman and Brunk, 2004; Kurz et al., 2008) as well as a denatured form of H-ferritin (Miyazaki et al., 2002). Hydrogen peroxide, a by-product of mitochondria respiration, levels of which increase with age, may diffuse into iron-laden lysosomes and enhance lipofuscin formation, with lipofuscin possibly providing a catalytic surface for the Fenton reaction. The low pH and highly reductive environment of the lysosome favors rapid reduction of Fe^3+^ to Fe^2+^ (Schafer and Buettner, 2000; Kurz et al., 2011). Mitochondria also contain ferritin which can undergo autophagocytic degradation to provide an additional source of free iron to further enhance production of ROS (Eaton and Qian, 2002). The accretion of iron-catalyzed oxidative stress induces peroxidative damage of lysosomes and membrane permeability is increased, leading to iron leakage which in turn augments the labile iron pool (Eaton and Qian, 2002). Oxidative stress initiates a cascade of events, culminating in the formation of lipofuscin-containing aggresomes.

### Mitochondrial ferritin

Mitochondrial ferritin displays high sequence homology to cytosolic H-ferritin (Levi et al., 2001) and helps to regulate iron distribution between the cytosol and mitochondria to attenuate ROS-generation. Mitochondrial ferritin has been associated with an anti-oxidative (neuroprotective) effect in neurodegenerative diseases including AD and PD cells (*in vitro*) and mouse models (Wu et al., 2013; Arber et al., 2016; Guan et al., 2017). Ten-month old mitochondrial ferritin knock-out (KO) mice infused with Aß_25−35_ had exacerbated learning and memory impairment compared to wild-type counterparts, and augmented apoptosis (reflected by decreased Bcl-2/Bax ratio) in the hippocampus (Wu et al., 2013; Wang et al., 2017). The increase in neuronal apoptosis was concomitant with augmented hippocampal levels of L-ferritin and Fpn but decreased TfR1 expression (Wang et al., 2017). It is likely that inhibition of IRP binding underlies the reported biochemical changes due to an increased regulatory iron pool in neurons challenged with Aß (Lane et al., 2015). The levels of malondialdehyde (MDA), a product of lipid peroxidation and a surrogate for oxidative stress were also significantly increased (Wang et al., 2017). Remarkably, overexpression of mitochondrial ferritin restored concentrations of iron and its metabolizing proteins, reduced oxidative damage via the p38 mitogen-activated protein kinase (MAPK) and activated extracellular signal-regulated kinase (Erk) signaling (Wu et al., 2013). These findings point to mitochondrial ferritin being pivotal for labile iron pool regulation to limit oxidative stress and neurodegeneration in aging and AD (Valko et al., 2016).

### Iron dyshomeostasis and neuroinflammation

The neuroglia (astrocytes and microglia), play an essential role in maintaining brain iron homeostasis to modulate neuronal activity and regulate neuroinflammation (Pelizzoni et al., 2013). Iron has been shown to stimulate microglia, increasing activation of NFκB and production of pro-inflammatory cytokines (Saleppico et al., 1996). Ferrocene supplementation led to 1.4-fold increase in ferritin transcripts predominantly in the microglia and oligodendrocytes of organotypic hippocampal slices, accompanied by significant loss of olig2-positive cells and increased activated microglia (Healy et al., 2016). Increased release of IL-1ß and TNFα by iron-laden microglia induced upregulation of IRP1, DMT1, hepcidin and TfR1-1 expression in ventral mesencephalic neurons via production of ROS (Xu et al., 2016).

Conversely, neuroinflammation induced by stereotactic injection of LPS into the striatum, not only induced microglial activation but also ferritin expression and total nigral iron content in aged rats (Hunter et al., 2008). Interestingly, cytokine treatment (TNFα, IL-6 and LPS) stimulated DMT1 expression and accompanying downregulation of Fpn expression in neurons, astrocytes and microglia (Urrutia et al., 2013). Hepcidin secreted by astrocytes and microglia seems to be the phenomenon underlying inhibition of Fpn expression under conditions of inflammatory milieu. The cumulative effect of these changes is iron accumulation in neurons and microglia but not in astrocytes (see below). Deferoxamine reversed LPS-induced downregulation of Fpn and increased ferritin in the hippocampus (Zhang et al., 2015). Accordingly, deferoxamine has been shown to attenuate cognitive deficits in AD (Crapper McLachlan et al., 1991) and PD subjects (Ward et al., 2014). Moreover, deferoxamine counteracted LPS-induced hippocampal microglial activation and associated pro-inflammatory (TNFα and IL-1ß) cytokine production, caspase-3 mediated apoptosis and ameliorated memory impairment in mice (Zhang et al., 2015). Iron appears to have a role in the upregulated MDA expression (increased ROS) and downregulated SOD activity (decreased antioxidant activity) in LPS-induced neuronal injury and cognitive impairment. Iron can activate GSK-3ß, a downstream target of Akt involved in regulation of important cellular brain processes and is a positive regulator of inflammation (Martin et al., 2005; Zhang et al., 2015). Overexpression of GSK-3ß leads to apoptosis and a decline in the number and volume of postsynaptic hippocampal granular neurons, changes putatively linked to cognitive impairment. Interestingly, intracerebroventricular injections of LPS reduced the phospho-GSK3ß/GSK3ß ratio in the hippocampus resulting in abnormal activation of GSK-3ß (Zhang et al., 2015). Deferoxamine was able to attenuate GSK-3ß phosphorylation, in turn downregulating caspase-3 mediated apoptosis in the LPS-exposed mouse brains. On a different note, deferoxamine has been shown to downregulate levels of NADPH oxidase subunits (gp91phox) which are able to precipitate oxidative stress (Li et al., 2016). Also, deferoxamine can inhibit p38 MAPK activation and is associated with reductions in pro-inflammatory cytokines. Anti-inflammatory drugs had a similar effect to chelation therapy, reversing iron-induced synaptic loss, apoptosis, and impairment of mitochondrial dynamics in rodents exposed to iron overload (Nagarkatti et al., 2009; da Silva et al., 2014; Lavich et al., 2015).

The positive feedback loop between neuroinflammation and iron accumulation contributes in a progressive manner to promote neurotoxicity. Based on the precept that iron dysregulation enhances ROS generation and cellular damage, alterations in proteins involved in iron regulation amplifies iron-dependent oxidative stress contributing to neurodegeneration. The different lines of evidence emphazise the intimate relationship between iron accumulation, neuroinflammation and cognition in physiological and detrimental aging.

Astrocytes appear to be more resilient to iron accumulation owing to their GPI-anchored ceruloplasmin, responsible for oxidizing Fe^2+^ to Fe^3+^, enabling Fpn-mediated cellular iron export (De Domenico et al., 2007). GPI-ceruloplasmin aids localization of Fpn to the cell membrane for optimal functioning (De Domenico et al., 2007). The importance of ceruloplasmin in regulating the labile iron pool and contributing to anti-oxidant defense is underscored by findings in aceruloplasminaemia subjects. The GPI-ceruloplasmin deficiency in aceruloplasminaemia impairs cellular iron egress, resulting in widespread brain iron accumulation particularly in the basal ganglia leading to neurodegeneration and motor deficits (Xu et al., 2004; Ward et al., 2014).

Experimental evidence suggest the choroid plexus has the capacity to sequester metals including iron as an essential CNS defense mechanism (Zheng, 2001). The epithelial cells of the choroid plexus synthesize iron-associated proteins including ceruloplasmin (Aldred et al., 1987). Tf is also secreted by the choroidal epithelial cells (Aldred et al., 1987), which may bind Fe^3+^ and drain iron from the CSF possibly via the glymphatic system. Intriguingly, the choroid plexus from older wild-type mice and human post-mortem samples demonstrated increased transcript expression of type I interferons, i.e. upregulated neuroinflammation (Baruch et al., 2014). CSF acquired from 22-month old wild-type mice, used to incubate primary cultures of young (3-month old wild-type mice) choroidal epithelial cells, augmented type I interferon transcripts. CSF injection of antibodies against the type I interferon receptor in aged mice rescued memory deficits, attenuated neuroinflammation and restored neurogenesis to the levels seen in young mice (Baruch et al., 2014). Apparently, the choroidal epithelium undergoes dynamic changes during aging and dementia with reduced ability of the choroid plexus to regulate brain interstitial fluid (Serot et al., 2003; Wyss-Coray, 2016), promoting a pathological oxidative CSF environment. Accordingly, the ferroxidase activity of soluble ceruloplasmin in the CSF is significantly attenuated in PD patients (Barbariga et al., 2015). Ceruloplasmin oxidation promotes asparagine deamidation, a spontaneous reaction that happens during protein aging to form aspartate and iso-aspartate. Deamination of specific asparagine-glycine-arginine motifs of ceruloplasmin are in turn able to attenuate ceruloplasmin activity. Protein deamination is associated with greater integrin-binding and intracellular signaling (Erk1/2, Akt and MAPK) that may contribute to iron dysregulation via modified gene activation, cell proliferation and re-arrangement of the actin cytoskeleton (Barbariga et al., 2015). Interestingly, deamidation-mediated protein aggregation has been reported for α-synuclein in PD (Vigneswara et al., 2013), Aβ and tau in AD (Shimizu et al., 2000). The oligomers in combination with iron dyshomeostasis may propagate disease further in PD and AD. Idiopathic PD subjects showed ~80% loss of ceruloplasmin ferroxidase activity in the substantia nigra, which contributes to the accumulating pro-oxidant iron characterizing PD pathology (Ayton et al., 2013). As proof of concept, peripheral infusion of ceruloplasmin attenuated nigral iron accumulation and neurodegeneration in the 1-methyl-4-phenyl-1,2,3,6-tetrahydropyridine (MPTP) mouse model for PD. Also, ceruloplasmin-knockout mice developed Parkinsonism that was reversed by iron chelation (Ayton et al., 2013). It is important to emphazise that CSF/plasma levels of ceruloplasmin (and ferritin) may not be altered in PD and AD but their functional status may be altered (Kristinsson et al., 2012).

Adult ceruloplasmin null-mice demonstrate increased iron deposition in widespread regions of the brain including in the cerebellum and brainstem (Cui et al., 2009). The striatum characteristically exhibits iron accumulation in subjects with aceruloplasminaemia but shows normal iron levels in ceruloplasmin KO mice. This discrepancy perhaps reflects the shorter lifespan of mice compared to humans. More importantly, the regional variability in iron load in ceruloplasmin null-mice is due to hephaestin expression, another multi-copper oxidase, and a homolog of ceruloplasmin. Hephaestin levels are significantly elevated in the cerebral cortex and striatum of 80-week old ceruloplasmin KO mice relative to wild-type mice (Cui et al., 2009). On the other hand, hephaestin expression was slightly attenuated in the substantia nigra and cerebellum with relatively unchanged levels in the hippocampus. Apparently, the augmented hephaestin levels compensates for the loss of ceruloplasmin so as to maintain normal iron concentrations in select regions such as the cerebral cortex and the striatum. Hephaestin KO mice exhibit significantly increased iron and L-ferritin in the mouse brain cortex, hippocampus, brainstem, and cerebellum compared to ceruloplasmin KO and wild-type at 6 months of age (Jiang et al., 2015). Ablation of the multi-copper oxidases, ceruloplasmin, and hephaestin, leads to deregulated brain iron homeostasis in mice and present useful models for exploring neurodegenerative diseases (Cui et al., 2009; Jiang et al., 2015).

## Neuroimaging perspective on iron deposition

The advent of neuroimaging has provided the unprecedented opportunity to study age-related changes *in vivo*, and evaluate efficacy of therapeutic strategies. Paramagnetic properties exhibited by iron render magnetic resonance imaging (MRI) a promising tool to evaluate brain iron content as iron deposition induces changes in the transverse relaxation time (T2) (Daugherty and Raz, 2015). Iron can also induce local magnetic susceptibilities and reduce T2^*^. Histological data suggests ferritin and hemosiderin (and neuromelanin in substantia nigra) are the only paramagnetic agents expressed at sufficient concentrations to affect the brain MRI signal (Schenck, 1995). Estimated binding of non-heme iron to ferritin in physiological conditions is 90%, while concentrations of Tf-bound iron or the putatively pathogenic pool of labile free iron is low (Moos, 2002; Haacke et al., 2005).

Meta-analysis of twenty MRI studies involving healthy aged subjects reported similar age-related iron changes in the globus pallidus to post-mortem findings (Daugherty and Raz, 2013). Though iron deposition was found to be elevated in subcortical nuclei, the globus pallidus emerged with the highest iron content with age, preceded by the striatum, red nucleus, and substantia nigra (Acosta-Cabronero et al., 2016). Cross-sectional neuroimaging studies suggest a significant, albeit smaller, positive correlation between age and iron deposition in the thalamus and hippocampus. Much of the age-related trajectory evidence has originated from cross-sectional data and may not capture the dynamics of the intricate aging process. On the other hand, longitudinal assessments can help evaluate the key role of a plethora of mediators in age-related associations between brain and cognitive performance. Using R2^*^ (reciprocal of T2^*^) relaxometry, a longitudinal study demonstrated higher baseline iron deposition in the striatum predicted worsening of working memory after 2 years (Daugherty et al., 2015). This finding was replicated in an independent sample of older adults with multiple measurements performed over a period of 7 years (Daugherty and Raz, 2015, 2016). Potentially, brain iron deposition may be a biomarker of cognitive decline in normal aging.

Increased R2 (reciprocal of T2) and R2^*^ signal (reflective of iron accumulation) was observed in mice during the course of normal aging (2–27 months) in the basal ganglia (Walker et al., 2016). R2 and R2^*^ correlated well with iron content measured using synchrotron-based X-ray fluorescence iron mapping. The iron accumulation was concomitant with increased ferritin immunoreactivity in all basal ganglia regions except the substantia nigra. The discordance between iron and ferritin upregulation is indicative of iron dyshomeostasis in the aged substantia nigra. Moreover, nigral neurons has been shown to be more susceptible than hippocampal or cortical neurons to LPS-induced neurodegeneration and oxidative stress (Bartzokis et al., 2004; van Rooden et al., 2015). Furthermore, the substantia nigra, already abundant in microglia, exhibits microgliosis, and astrogliosis with aging. The iron dysregulation, alongside microgliosis, and astrogliosis, as well as the known high metabolic activity of nigral neurons (Crichton et al., 2011; Ward et al., 2011; Walker et al., 2016), together may explain the particular vulnerability of the aged substantia nigra to neuropathology. Interestingly, dietary restriction (intermittent fasting), a robust “anti-aging” regimen in rodents, attenuated age-related *in vivo* R2 increases in the substantia nigra of aged mice suggesting some restoration of age-associated iron dysregulation (Walker et al., 2016).

### AD

A longitudinal MRI study conducted in a transgenic mouse model of amyloidosis (12–18 months) found increased amyloid plaque formation coincided with an augmented R2 in the hippocampus and cerebral cortex (Braakman et al., 2006). This observation is consistent with MRI human findings, where elevated iron levels in the hippocampus were negatively correlated with cognitive performance assessed by the mini-mental state examination (MMSE) (Zhu et al., 2009; Raven et al., 2013; Langkammer et al., 2014). Interestingly, iron is associated with Aß plaque formation and a constituent of core and halo plaques (Everett et al., 2014), with Fe^3+^ bound to Aß being reduced to Fe^2+^ and promoting ROS production. The pro-oxidant environment escalates the production of Aß oligomers/plaques via enhanced amyloidogenic processing of APP (mediated by ß-secretase) (Everett et al., 2014; Peters et al., 2015). An *in vivo* study demonstrated iron overload in APP/PS1 (amyloidosis) mouse model stimulated amyloidogenic processing and altered neuronal signaling to increase Aß plaque formation, leading to cognitive deterioration (Becerril-Ortega et al., 2014). Although some report unaltered total brain iron contents in AD, nonetheless, microscopic focal iron deposits are found either in the plaque core or in the vicinity (Langkammer et al., 2014). PET-ligand ([^11^C]-PIB), specific to fibrillary Aß plaques (Nordberg, 2004), was found to be significantly associated with higher cortical iron (as revealed by quantitative susceptibility mapping which is sensitive to iron-induced changes in susceptibility) in Aß-positive cognitively normal, MCI and AD (van Bergen et al., 2016; Ayton et al., 2017b), and synergistically exacerbated cognitive performance. This is consistent with increased iron deposition coinciding with early plaque formation in a mouse model of AD (Leskovjan et al., 2011). High CSF ferritin (used as a proxy measure for iron load) was significantly associated with accelerated depreciation of CSF Aß, implying facilitation of Aß deposition in AD (Ayton et al., 2017a). Strikingly, the high CSF ferritin progressed transition from threshold preclinical Aß levels to the average level of AD from approximately eighteen to 10 years. The different lines of evidence suggest that disease progression is conferred by spatial colocalization of iron with Aß deposition, and confirms an association between increased brain iron load and AD.

The onus is being placed on identifying the cellular localization of iron to investigate which cells (glia or neuron) specifically accumulate iron with aging. Adopting this approach, focal MRI hypo-intensities were identified in the hippocampus, predominantly in the subiculum of AD subjects at post-mortem (Zeineh et al., 2015). Co-registration of *ex vivo* MRI with histology enabled localization of the MRI-detected iron deposits to (CD163-identified) activated microglia. The subiculum is an important structure via which entorhinal perforant and alvear pathway neurons travel to the hippocampus. The identified iron deposition associated with microglial activation may augment entorhinal neurodegeneration, known to be affected early on in AD. The microtubule associated protein tau is also observed in the subiculum. These findings provide an incentive to monitor progression of iron deposition and neuroinflammation with particular relevance to their association with Aß and tau pathology.

Total levels of CSF ferritin were unaltered when comparing cognitively normal, MCI, and AD subjects in the Alzheimer's disease neuroimaging initiative (ADNI) cohort (Ayton et al., 2015a). Nevertheless, CSF ferritin was shown to predict cognitive outcomes over a period of 7 years. MCI subjects with high ferritin levels demonstrated an earlier age of diagnosis (Ayton et al., 2015a), consistent with previous reports, where an earlier age of onset of AD is associated with greater neocortical iron burden as assessed by MRI *in vivo* (Bartzokis et al., 2004; van Rooden et al., 2015). In addition, high CSF ferritin load in MCI subjects was associated with MRI-assessed hippocampal atrophy and AD biomarkers (Ayton et al., 2015a). It would be interesting to determine the association of other CSF proteins involved in iron metabolism (including ceruloplasmin, transferrin) and evaluate them as predictors of conversion of subjects to AD.

### PD

PD is characterized by classical iron accumulation in the substantia nigra and basal ganglia that appear hypointense on a T2^*^-weighted MRI *in vivo* (Pyatigorskaya et al., 2014). The increased iron content observed in the aforementioned regions correlates with severity of cognitive and motor impairments (Atasoy et al., 2004). Likewise, a new genetic mouse model of PD, demonstrated reduced T2^*^ of the substantia nigra and striatum indicative of abnormal iron load and associated with motor and cognition deficits (Cong et al., 2016). Interestingly, augmented iron load (reduced T2^*^) in the substantia nigra preceded motor symptoms in PD subjects (Martin et al., 2008; Wallis et al., 2008; Du et al., 2011; Ulla et al., 2013; Aquino et al., 2014; Devos et al., 2014; Hopes et al., 2016). Iron accumulation increased rapidly over the first 3–5 years of PD but progressed much more insidiously in advanced stages (Hopes et al., 2016). Iron overload showed a higher degree of correlation with motor symptom severity at earlier stages of the disease, while lack of correlation was observed at later stages of PD. MPTP-intoxicated mice exhibited iron overload (decreased T2^*^) only 7 days after MPTP administration, recapitulating the results obtained in PD patients (Hopes et al., 2016). In another study, increased nigral iron content precipitated oxidative stress and dopaminergic neuronal death in a MPTP-induced PD mouse model, further reinforcing T2^*^ as an early, sensitive biomarker with translational potential (You et al., 2015). Moreover, iron content in the substantia nigra and locus coeruleus is substantially increased in PD subjects, and loss of nigral neurons in particular leads to a substantial reduction of neuromelanin (Wang et al., 2016a). Since neuromelanin can chelate iron and thought to prevent toxic accumulation of iron (Zecca et al., 2008b), a reduction in neuromelanin content may explain the increase in labile iron pool. The free iron may exceed the iron-binding capacity of ferritin as well as bind to Lewy bodies (Castellani et al., 2000), increasing neuronal and glial toxicity via oxidative stress (Griffiths et al., 1999).

Recently, a handful of studies have demonstrated MRI T1-weighted fast spin echo appeared to identify neuromelanin (Sasaki et al., 2006). The hyperintense T1-signal of the substantia nigra corresponded histologically to neuromelanin-containing neurons (Kitao et al., 2013). In PD, this neuromelanin signal in the substantia nigra and locus coeruleus attenuates with disease progression (Schwarz et al., 2011; Ogisu et al., 2013; Reimão et al., 2015). A greater reduction in T1-weighted signal was also observed in the lateral segment of the substantia nigra with relatively preserved medial aspect, in agreement with the reported pathological characteristics of neuronal atrophy in PD (Miyoshi et al., 2013). MRI-detected neuromelanin measurements have demonstrated high sensitivity and specificity for differentiating between healthy controls and PD subjects (Miyoshi et al., 2013). MRI offers high diagnostic accuracy also at early stages of disease, only slightly less than with [^123^I]-FP-CIT-SPECT studies. A greater reduction in the neuromelanin signal is observed in advanced PD compared to early stages (Schwarz et al., 2011). A gradual and stage-dependent decrease in neuromelanin in the medial substantia nigra was indicated by T1-weighted MRI in PD subjects, with pronounced reduction of neuromelanin in the locus coeruleus in later stages, suggesting alterations commence ventrolaterally, and extend medially in PD. Thus, MRI measurements, thought to be of neuromelanin, may be useful for monitoring disease progression.

The tau tangle PET ligand, [^18^F]-AV-1451 ([^18^F]-T807), exhibits off-target binding to neuromelanin in the midbrain, and is proposed as a measure of the pigmented dopaminergic neuronal count in the substantia nigra (Hansen et al., 2016). Interestingly, off-target AV-1451 binding patterns (using autoradiography) observed in the basal ganglia correspond with Perl's staining, suggesting AV-1451 associate with iron. High AV-1451 binding in the substantia nigra is consistent with the high iron content in this region. The high binding avidity of AV-1451 to the substantia nigra, even in subjects with no tau pathology, argues for its potential binding to iron (Lowe et al., 2016). However, significant Perl's staining for iron evident in the globus pallidus of a NBIA case did not coincide with AV-1451 binding pattern, suggesting that the off-target moiety for this tracer is neuromelanin (Lowe et al., 2016). Future cross-validation MRI-PET studies are warranted to validate the prognostic value of neuromelanin-sensitive MRI and AV-1451.

## Genetic regulation of iron-associated proteins

**Figure 5:**
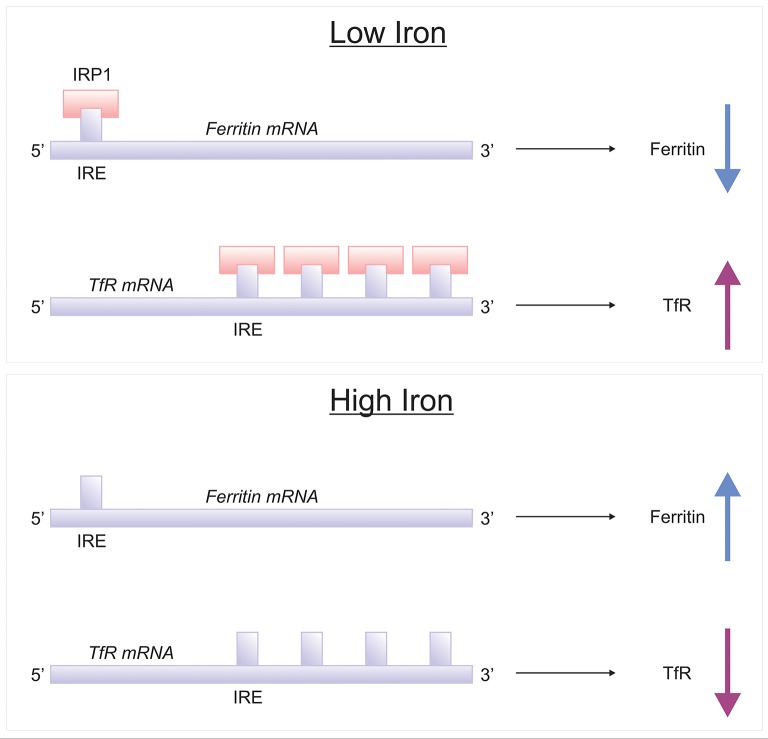
IRE/IRP regulation of ferritin and transferrin receptor. Proteins involved in the storage, export and uptake of iron are regulated via the interaction of iron-regulatory proteins (IRPs) with iron-responsive element (IREs), conserved RNA secondary structures (Rouault, 2006; Leipuviene and Theil, 2007). In conditions of low iron, IRP binds to a single IRE in the 5′-untranslated region (UTR) of ferritin mRNA to suppress their translation, while IRP binding to multiple IREs in the 3′ UTR of transferrin receptor-1 (TfR1) which stabilizes the mRNA for TfR1 synthesis. When the iron content in the cell is high, the lack of IRP-binding leads to increased synthesis of ferritin and destabilization of TfR1 mRNA.

Brain iron homeostasis is regulated by IRPs, IRP1, and IRP2 (Rouault, 2006) (Figure 5). When cellular iron is high, IRP bind to the IRE of transferrin receptor and DMT1 mRNAs, decreasing their translation, resulting in attenuated cellular uptake of iron. Conversely, when cellular iron levels are low, IRPs binds to the IRE situated at the 3′-UTR of transferrin receptor and DMT1 mRNAs, stabilizing them and increase their cellular expression level, augmenting iron uptake. Also IRP bind to IREs of ferritin and Fpn mRNAs, rendering them unstable and lowering protein expression (Leipuviene and Theil, 2007), effectively preventing iron sequestration by ferritin or cellular iron export by Fpn, maintaining iron homeostasis. Interestingly, mice with genetic ablation of IRP1^−/−^ do not exhibit neurodegeneration as IRP1 minimally contributes to iron regulation in the wild-type (LaVaute et al., 2001). However, IRP2-null mice demonstrate significant iron deposition in white matter tracts and many brain nuclei, particularly in the striatum, thalamus, cerebellum and colliculi. Ferritin (iron) accumulates abnormally in neuronal and oligodendroglial populations particularly in the striatum and the thalamus (LaVaute et al., 2001). The severe iron dyshomeostasis leads to neurodegeneration in adulthood followed by development of motor symptoms in the form of ataxia, bradykinesia and tremor (LaVaute et al., 2001; Smith et al., 2004). Interestingly, pronounced worsening of the neurodegeneration was evident in IRP1^+/−^ IRP2^−/−^ mice relative to IRP1^+/+^ IRP2^−/−^ mice, suggesting loss of one IRP1 allele further aggravates iron metabolism, and predominantly affecting the substantia nigra. Additionally, activated microglia were associated with neurodegeneration, implying a role for iron deposition in fuelling neuroinflammation (Smith et al., 2004).

The interesting finding of APP and prion protein (PrP) as physiological regulators of iron homeostasis puts AD and prion diseases in the limelight (Singh et al., 2009; Singh, 2014). Iron putatively modulates APP processing via interaction of IRP with IRE in the 5′-UTR of its mRNA, positioned immediately upstream of an interleukin-1 responsive acute box domain (Rogers and Lahiri, 2004; Bandyopadhyay and Rogers, 2014). Under conditions of low iron as observed with the use of iron chelators (Rogers et al., 2002), IRP1 may bind to the IRE of APP with high affinity to repress APP translation. Conversely, high iron load likely upregulates APP translation and increases the amyloidogenic processing of APP yielding Aß species. Meanwhile, increased interleukin-1 may increase IRP binding to APP decreasing APP production (Rogers and Lahiri, 2004; Bandyopadhyay and Rogers, 2014). Interestingly, decreased APP expression in nigral dopaminergic neurons of human PD cases was observed, and likewise APP-knockout mice developed iron-mediated nigral cell loss (Ayton et al., 2015b). The augmented nitric oxide (NO) was shown to suppress APP translation in MPTP PD mouse models pinpointing NO toxicity as a mechanism for causing iron-dependent neurodegeneration in PD. On the other hand, APP-overexpressing mice were immune to MPTP toxicity. Moreover, a putative IRE in the 5′-UTR of mRNA encoding the 140-residue α-synuclein has been reported (Friedlich et al., 2007), suggesting that this RNA structure post-transcriptionally controls α-synuclein production in response to cellular iron.

APP has been suggested to possess ferroxidase activity to aid cellular iron export but supporting evidence is lacking. APP knockout mice demonstrate markedly increased iron in the brain and several other organs (Duce et al., 2010). It is likely that the neuroprotective properties of APP are conferred by soluble APPα, a cleavage product of non-amyloidogenic pathway, as a point mutation within the REXXE motif nullifies its neuroprotection properties (Duce et al., 2010). Emerging evidence suggests that the endogenous APP stabilizes Fpn in the neuronal membrane, supporting its role in iron export from neurons (Wong et al., 2014).

Interestingly, IRP-IRE binding activities together with ferritin were found to be increased in the astrocytes of hippocampus and cerebral cortex in PrP^Sc^ (scrapie form of PrP, a ß-sheet rich insoluble conformer) mice (Kim et al., 2007). Moreover, PrP^c^ (native form of PrP) is a ferrireductase, facilitating influx of iron by TfR1 and enhancing non-Tf bound iron uptake via DMT1 and the Zip Fe/Zn transporter (latter, independent of TfR1 activity). PrP^c^ is highly expressed in neurons and low in glial cells (Singh et al., 2009). Taken together, iron dysregulation appears to be a common theme prevalent in the etiology/pathogenesis of neurodegenerative diseases.

## Effect of systemic iron overload on brain iron

Hereditary hemochromatosis caused by mutations in the HFE gene (C282Y and H63D being the most common polymorphisms) is characterized by excessive dietary iron absorption and accumulation in various organs of the body (Nandar and Connor, 2011). Most particularly, liver iron deposition causes fibrosis and cirrhosis (Banerjee et al., 2014) but the effect of systemic iron on the brain remains elusive. HFE is a membrane protein implicated in controlling iron absorption via regulation of the affinity of TfR1 for holo-Tf. Although a link between HFE mutation and iron abnormalities in the framework of developing AD has been postulated (see below), studies investigating the brain effects of HFE dysfunction are sparse in humans. However, some important advances have been made using HFE knockout and knock-in mouse models.

Male HFE knockout mice at 3 months of age experienced motor coordination deficits but these behavioral manifestations were not paralleled by brain iron accumulation (Golub et al., 2005). Also, rat models of dietary iron overload demonstrate behavioral disturbances without obvious elevation in brain iron (Sobotka et al., 1996). Likewise, an H67D knock-in mouse line (equivalent to human homology of H63D) did not exhibit significant increases in total brain iron concentration but iron-related proteins were significantly changed (Nandar et al., 2013). Increased expression of HFE and H-ferritin was observed in 6-month old H67D mice, alongside astrogliosis, increased protein oxidation, expression of ${\text{x}}_{\text{c}}^{-}$ and HO-1, suggestive of heightened metabolic and oxidative stress. Accordingly, the striatum of HO-1 knockout mice was protected from iron-mediated injury in males but not females (Wang et al., 2016b). Meanwhile, H67D mice at 12-months had augmented H- and L-ferritin accompanied by attenuated Tf expression, indicating increased storage of iron (Nandar et al., 2013). The accruing oxidative stress is attenuated by increased iron storage in microglia (L-ferritin positive). Interestingly, no astrogliosis or oxidative stress was observed at 12-months, attenuation of the latter process may be explained by significant increments in nuclear factor E2-related factor 2 (Nrf2) and compensatory increased expression of anti-oxidant enzymes. Furthermore, DMT1 levels were also significantly decreased in 6-month old H67D, lower in the cortex, striatum, and cerebellum compared to wildtype mice (Nandar et al., 2013). DMT1 is responsible for endosomal iron export, its downregulation decreases cellular iron availability. At the cellular level, human neuronal cell lines harboring H63D polymorphism demonstrated a higher labile iron pool, increased oxidative stress, altered glutamate homeostasis, chemokine secretion, and tau hyperphosphorylation (Nandar and Connor, 2011). Indeed, the H63D polymorphism is frequently observed in AD subjects (Sampietro et al., 2001; Ali-Rahmani et al., 2014) and is considered a risk factor for neurodegenerative diseases.

Systemic iron overload levels may indirectly affect the brain. For instance, high systemic iron can cause severe liver damage resulting in accrual of toxic substances such as ammonia in the blood. Ammonia being a toxic gas, freely diffuses across the BBB and hyperammonaemia leads to speech and motor impairment, seizures and coma (Bridle et al., 2003; Eroglu and Byrne, 2009; Cordoba, 2014). Interestingly, the importance of ammonia is underscored by its excessive formation in brains of AD subjects, and elevation of blood ammonia concentrations (Seiler, 2002). Ammonia exerts neurotoxicity via modulation of neuroinflammation, mitochondrial dysfunction, altered glutamate/GABA neurotransmission, these perturbations are manifested clinically in the form of cognitive decline (Adlimoghaddam et al., 2016). Phenylbutyrate, an ammonia quencher, was able to rescue learning deficits due to clearance of intraneuronal Aß and restoration of dendritic spine densities of hippocampal CA1 pyramidal neurons to control levels in Tg2576 mice (Ricobaraza et al., 2012). Likewise, phenylbutyrate protected dopaminergic neurons in the MPTP mouse model of PD via anti-inflammatory and anti-oxidant activity (Roy et al., 2012).

Iron overload has been shown to cause low-grade inflammation in the liver, and resulting alterations in circulating cytokines and immune cells may affect the brain without increases in total brain iron (Bridle et al., 2003; Eroglu and Byrne, 2009; Cordoba, 2014). Indeed, systemic inflammation has been postulated to be involved in the pathogenesis of neurodegenerative diseases, by further exacerbating neuroinflammation (Perry et al., 2003).

A number of studies have evaluated alterations in brain iron concentration in response to iron supplementation at various ages. Neonatal excess iron supplementation augmented iron load in the basal ganglia at 3-month old mice, accompanied by impaired performance in radial arm maze learning tests and had poor spontaneous motor behavior (Schröder et al., 2001). Impaired recognition memory was also observed and reversed by selegiline, a monoamine oxidase inhibitor, and desferoxamine. Administration of iron as early as postnatal days 5–7, or as late as days 19–21 in rats, induced oxidative stress in the hippocampus, cortex, and substantia nigra (Dal-Pizzol et al., 2001). Furthermore, iron administration for three consecutive days (postnatal days 12–14) in neonatal rats led to increased apoptotic markers (caspase-3 and Par4) in the hippocampus and cortex of adult rats (Miwa et al., 2011). GFAP-labeled astrocytosis was increased in the hippocampus of 3-month old rats treated with iron between postnatal days 12 and 14, and in the substantia nigra and striatum of 2-year old iron-treated rats (Fernandez et al., 2011). The prevailing evidence suggests that dietary iron supplementation early in neonatal period is associated with cellular imprinting in the brain at a later stage in life.

Male weanling rats fed an excess iron supplemented diet for 6 or 8 weeks demonstrated significant iron load in the cortex, hippocampus, substantia nigra, and striatum (Chang et al., 2005; Ke et al., 2005; Qian et al., 2007). Moreover, 8 week supplementation with ferrocene from aged 4-weeks in male and female mice, led to an 8-fold increase in liver iron (Malecki et al., 2002). L-ferritin and HO-1 (indicators of increased iron) were elevated in the striatum while the iron concentration was increased in the cerebrum. Intraperitoneal injection of Fe^2+^ sulfate in adult rats for five consecutive days resulted in impaired emotional behavior and spatial learning (Maaroufi et al., 2009), with associated with significant iron accumulation in the hippocampus and basal ganglia.

In summary, small rodent studies have shown that peripheral iron administration/excess iron supplementation may lead to iron accumulation in the brain and mediate brain dysfunction via oxidative stress, induced neuroinflammation and apoptosis, and associated with behavioral impairments.

## Cellular brain barriers: first line of defence

The BBB is known to be established and functional during embryogenesis in both humans and rodents, specifically embryonic day 15.5 for mice (Ben-Zvi et al., 2014). The tight junctions in cerebral vessels and choroid plexus are formed early in embryonic development, however, the capillaries within the rodent cortex only begin to appear adult-like during postnatal days 14–21 (Semple et al., 2013). This is because the main period of astrocytic differentiation and the increasing coverage of capillaries with astrocytic end-feet occurs in rodents in the first 3-weeks of life (Caley and Maxwell, 1970; Saunders et al., 2014). This explains the significant brain iron overload (and associated behavioral impairments) following neonatal administration of iron in rodents. Thus, a compromised BBB may play a putative role in aging and neurodegenerative diseases. For instance, cerebral vasculature inflammation is regarded as an early event in the progression of neuroinflammation and Aß pathology (Biron et al., 2011). BBB inflammation has been observed with alterations in permeability and upregulation of MECA-32 and selectin in rodent models of neurodegeneration (Yu et al., 2012). The activation of the endothelium has been shown to coincide with increased BBB permeability which may enable systemic inflammatory cells including monocytes/macrophages to enter the brain to initiate iron deposition. Systemic inflammation can induce BBB perturbations leading to increased microglial/astrocytic activation, iron deposition, protein aggregation and neurodegeneration (Hernandez-Romero et al., 2012; Urrutia et al., 2013; Andersen et al., 2014).

## Concluding remarks and future directions

Brain iron is tightly regulated by a multitude of proteins and cellular barriers, with perturbation in their metabolism underlying the observed iron dyshomeostasis during brain aging, putatively promoting oxidative cellular damage. Neuroimaging, particularly involving the use of MRI, offers a non-invasive quantitative approach to study brain aging. However, MRI is limited by the inability to measure concentrations and cellular/subcellular location of individual iron species. A multi-modal approach needs to be adopted, e.g., where MRI is used together with histological/molecular biology tools. The latter ascertaining the cellular localization (i.e., neurons, microglia, or astrocytes) of iron, and how expression of iron-regulatory proteins changes in healthy and pathological aging (or neurodegenerative disease) in respective compartments.

Iron dyshomeostasis is increasingly considered a hallmark of neurodegenerative diseases, where it is associated with neuroinflammation, protein aggregation, neurodegeneration, and neurobehavioural deficits (Ward et al., 2014). The exact mechanistic processes involved in normal and pathological aging in specific brain areas require further elucidation. To interpret the regional perturbations in iron and iron-related proteins, accurate iron mapping using multi-disciplinary approach in healthy aging and neurodegenerative disorders at different ages is required. Age and sex are important co-factors that need to be considered for dissecting pathology aging from healthy aging. Total subcortical iron levels were found to be lower in healthy women than men (Persson et al., 2015). Similarly, worse verbal-memory performance was associated with higher hippocampal iron in men but not women (Bartzokis et al., 2011). It is established that women have lower peripheral iron levels compared to men, consequently women have reduced brain iron levels (Whitfield et al., 2003). There is evidence to suggest that males who have a higher concentration of brain ferritin iron levels experience an earlier age of onset for acquiring neurodegenerative diseases including AD and PD (Bartzokis et al., 2004). Future studies should stratify subjects based on sex and genetic status to provide greater mechanistic insights in normal and pathological aging.

The recent deferiprone clinical trials in PD (Devos et al., 2014) and amyotrophic lateral sclerosis (Martin-Bastida et al., 2017; Moreau et al., 2017) were well tolerated and showed reductions in brain iron load, and was associated with improved clinical performance, and a better quality of life. However, there is increased incidence of agranulocytosis with deferiprone (Tricta et al., 2016) and there is a need to develop second generation of iron chelators without this side-effect (Xie et al., 2016). Based on the evidence discussed, we suggest a synergistic use of iron-chelation therapies (Hider et al., 2008) and/or anti-inflammatory treatments as putative anti-brain aging therapies to counteract pathological aging observed in neurodegenerative diseases.

## Author contributions

AA and P-WS conceived and designed the manuscript. All authors made significant contributions, reviewed and approved the manuscript.

### Conflict of interest statement

The authors declare that the research was conducted in the absence of any commercial or financial relationships that could be construed as a potential conflict of interest.
